# Emotion processing and electrodermal activity in young people who self-harm

**DOI:** 10.1038/s44220-025-00520-5

**Published:** 2025-11-05

**Authors:** Karen Wetherall, Seonaid Cleare, Nadia Belkadi, Marianne E. Etherson, Krystyna J. Loney, Susan Mathew, James Munro, Ellen Townsend, Matthew K. Nock, Eamonn Ferguson, Rory C. O’Connor

**Affiliations:** 1 Suicidal Behaviour Research Laboratory, School of Health and Wellbeing, University of Glasgow, Glasgow, UK; 2 Department of Child and Adolescent Psychiatry, Institute of Psychiatry, Psychology and Neuroscience, King’s College London, London, UK; 3 Faculty of Arts and Social Sciences, School of Psychology and Counselling, The Open University, Milton Keynes, UK; 4 School of Psychology, University of Nottingham, Nottingham, UK; 5 Department of Psychology, Harvard University, Cambridge, MA USA; 6 National Institute for Health and Care Research Blood and Transplant Research Unit in Donor Health and Behaviour, University of Cambridge, Cambridge, UK; 7 Pain Centre Versus Arthritis, University of Nottingham, Nottingham, UK

**Keywords:** Psychology, Physiology, Predictive markers, Risk factors

## Abstract

The biological underpinnings of self-harm in young people are unclear. Self-harm often serves to regulate emotions, and electrodermal activity (EDA) is a well-established biomarker of emotional arousal, which is physiologically related to emotion regulation. A quasi-experimental case control study using predefined groups was conducted. Three groups of young people (16–25 years; *n* = 180) with different self-harm histories were recruited: no self-harm history (*n* = 62), self-harm ideation last year with no enaction (*n* = 51) and self-harm enaction last year (*n* = 67). EDA was measured during three tasks: an auditory tones habituation task, a psychosocial stress task and an emotional images task. Those in the self-harm enaction group elicited a heightened EDA response (hyperreactivity) across two tasks, specifically a slower habituation rate to auditory tones and higher EDA during the psychosocial stress task compared to other groups. High levels of non-response during the emotional images task limited analyses. These findings expand our understanding of the biomarkers for self-harm, specifically emotional arousal in young people who self-harm. Specifically, they suggest that those with a history of self-harm exhibit a heightened electrodermal response to both stressful and non-stressful stimuli compared to those who have no history of self-harm and those who have only thought about self-harm.

## Abstract (web summary)

Young individuals with a history of self-harm exhibit heightened electrodermal activity in response to neutral and stress-related stimuli, extending our understanding of the biophysiological factors for self-harm vulnerability.

## Main

Self-harm, defined as intentional self-poisoning or self-injury irrespective of motive^1^, is relatively common in adolescents and young adults, with a meta-analysis of community studies finding up to 22% reported self-harm during their lifetime and 23% in the past 12 months^2^. For some young people, self-harm is an emotion-regulation tool used to control distress^3^, with an influential meta-analysis concluding that the most common function of self-harm was to modify the intensity or duration of emotions^4^, and a further qualitative meta synthesis suggesting self-harm helped to control overwhelming feelings^5^.

Self-harm is a complex behavior that stems from the interplay of social, psychological and biological mechanisms^6^. Much research has focused on the social and psychological factors, and these factors often guide psychosocial treatments for both adults^7^ and adolescents^8^. By contrast, the biophysiological mechanisms underlying self-harm have received limited attention, and these may improve our understanding of vulnerability to self-harm, as well as its onset and maintenance^9^.

Several biopsychosocial models conceptualizing the pathways to self-harm and suicidal behavior utilize an ideation-to-action framework^10^, distinguishing factors associated with self-harm ideation and self-harm enactment. Understanding the transition from thoughts to acts is essential to identify who is more likely to engage in self-harm. Factors that influence the transition from thoughts to acts of self-harm are described as volitional factors^11^, with growing evidence to support the ideation-to-action distinction^12,13^. Such studies have largely focused on psychological or psychiatric factors to the exclusion of physiological factors, an important omission given the emerging evidence that physiological activation linked to emotion arousal may be associated with mental health issues, self-harm and suicide attempts^14,15^.

Emotion regulation and emotional arousal are linked, with evidence suggesting that the brain systems involved in generating emotions (emotion arousal) and managing them (emotion regulation) are interconnected^16^. Specifically, the limbic system, including the amygdala, hippocampus and nucleus accumbens, is central to the processing of emotions, regulating mood, and the creation of emotional memories^17^. The amygdala may be particularly important for processing fear and heightened emotions, and the prefrontal cortex, used for cognitive control and executive function, is also active in regulating emotions^18^. Therefore, a focus on studying the biophysiological mechanisms underlying emotional arousal may be beneficial in relation to emotion regulation.

Electrodermal activity (EDA) has been proposed as a biomarker of emotional arousal, and, as it has been closely linked to autonomic emotional and cognitive processing, can be used to examine implicit emotional responses^19^. Specifically, EDA is a non-invasive measure of changes in the electrical conductance of the skin that depends on the quantity of sweat secreted by eccrine sweat glands, usually of the fingers or palms, which reflects the influence of the sympathetic nervous system. EDA is an overarching term with two components: skin conductance level (SCL), representing a slow tonic component reflecting general arousal, and skin conductance response (SCR), reflecting a faster phasic element of the signal appearing in reaction to the presentation of stimuli^20^.

A recent meta-analysis of the relationship between autonomic functioning and emotional dysregulation found that altered EDA was not directly associated with emotional dysregulation in young people, although it was noted that research was limited and additional studies were required^21^. Evidence indicates that EDA can be modulated by emotional regulation strategies^22^, suggesting that although EDA may not be a measure of emotion regulation, emotion arousal and emotion regulation may be physiologically linked. Therefore, dysregulated EDA, as a measure of sympathetic arousal, may be an important tool in understanding the underpinnings of self-harm.

So far, studies investigating the extent to which EDA is associated with self-harm and suicidal behavior have yielded mixed results^14,23,24^. For example, Nock and Mendes (2008) found young people with a self-harm history had increased reactivity (that is, higher tonic SCL) during a distressing task, implying they were hyperreactive (that is, experiencing heightened arousal)^14^. Other studies have found that individuals with a self-harm history exhibited lower SCRs, indicating hyporeactivity (that is, a dampened response) in response to stressful or emotional stimuli^23,25^. Furthermore, several studies have found no associations between SCL and self-harm^24,26,27^. It may be that these findings reflect the EDA measurement (that is, phasic SCR versus tonic SCL), the nature of the task and the different sample demographics. EDA may be affected by sex, age and self-harm history (ideation versus enaction). Indeed, a recent meta-analysis found that the type of self-harm may influence how the EDA dysregulation manifests; specifically, non-suicidal self-harm studies reported heightened EDA in young people who self-harmed, whereas those that included suicidal ideation and behaviors found decreased EDA^28^. There is also evidence to suggest that EDA may be sensitive to an individual’s physical state, for example, hydration levels, as well as environmental conditions such as temperature^19^.

Conflicting findings may also be related to the paradigm adopted to investigate dysregulated electrodermal responding; specifically, rather than a distressing or emotive task, impersonal stimuli may be used to elicit an autonomous SCR. Typically, after repetition of a stimulus such as a sudden tone, the SCR gradually reduces and then disappears as participants habituate^29^. A review has suggested that individuals who made a ‘violent’ suicide attempt (that is, attempted hanging or firearms) experienced EDA hyporeactivity (that is, fast habituation) to a sudden tone compared to non-suicidal individuals^15^. These findings suggest a potential psychobiological biomarker that may increase an individual’s capability to engage in suicidal behavior. Despite these promising findings, this method of eliciting EDA has not been tested with young people who self-harm, and the habituation of those who enact self-harm has not been compared to those who experience self-harm ideation only. Indeed, a recent meta-analysis^28^ found only studies that used an emotive or stressful stimuli. Therefore, the relationship between self-harm and EDA dysregulation in young people should be directly compared using different paradigms.

## Current study and hypotheses

To address these gaps in knowledge, we investigated the extent to which EDA distinguishes between young people who have different self-harm histories. Specifically, we compared EDA responses for three groups of young people aged 16–25 years: a control group (no history of self-harm), a self-harm ideation group (self-harm thoughts only) and a self-harm enaction group (enacted self-harm in the last 12 months). The study included four components with different stimuli and EDA measurements: (1) a baseline measure of tonic SCL and amplitude of non-specific skin conductance responses (NS-SCRs), (2) an impersonal tones habituation task (measure of the number of SCRs until habituation), (3) a psychosocial stress task (measure of tonic SCL during the task) and (4) viewing of emotive positive and negative images (measure of the average amplitude of SCRs).

We hypothesized that dysregulation of EDA (that is, being hyporeactive or hyperreactive) may act as a volitional factor facilitating the transition from thoughts of self-harm to self-harm acts^11^. Compared to those who have thought about self-harm (self-harm ideation group) and controls, those who have engaged in self-harm (self-harm enaction group) will show a dysregulated EDA response to different types of stimulus. Due to the previous inconsistent findings, we have not specified whether the dysregulation will be hypo- or hyperreactive (that is, blunted or heightened).

## Results

### Participant characteristics, psychiatric history and self-harm history

**Fig. 1:**
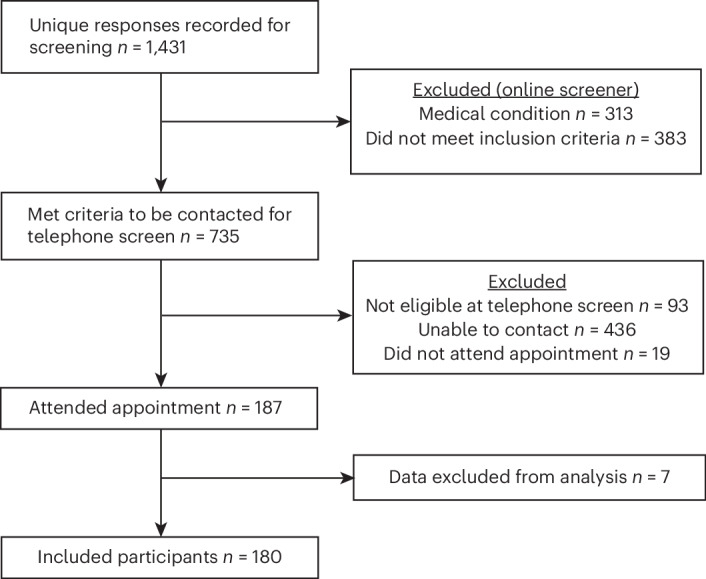
Study recruitment flow chart. A flow chart outlining participant exclusion and eligibility during the study recruitment.

A flow chart outlining study recruitment is outlined in Fig. 1. Table 1 outlines the participant characteristics for the self-harm groups. Of note, the enaction group had significantly more females (71.6%) than the control (50.8%) and self-harm ideation (56.9%) groups (*χ*^2^ = 6.576, *P* = 0.037). There were no significant differences between the groups by age (Fisher’s statistic *F*(2) = 0.748, *P* = 0.475). Age and sex were controlled for in all analysis.

**Table 1:** Participant characteristics for the self-harm groups and the total sample

	Control group (*n* = 62)	Self-harm ideation group (*n* = 51)	Self-harm enaction group (*n* = 67)	Total sample (*n* = 180)
Age, years^a^	21.37 (2.52)	20.98 (2.32)	20.85 (2.64)	21.12 (2.56)
Sex, % (*n*)^b^				
Female	50 (31)	56.9 (29)	71.6 (48)	60 (108)
Male	50 (31)	43.1 (22)	28.4 (19)	40 (72)
Ethnicity, % (*n*)				
Asian/British Asian	40.3 (25)	45.1 (23)	20.9 (14)	34.4 (62)
Black	3.2 (2)	2 (1)	0	1.7 (3)
Other^c^	11.3 (7)	13.7 (7)	16.4 (11)	13.9 (25)
White	45.2 (28)	39.2 (20)	62.7 (42)	50 (90)
Education, % (*n*)				
Degree/postgraduate	66.7 (42)	56.9 (29)	46.3 (31)	56.7 (102)
A levels/GCSE/NVQ	32.3 (20)	43.1 (22)	53.7 (36)	43.3 (78)
Employment, % (*n*)				
Student	82.3 (51)	80.4 (41)	76.1 (51)	79.4 (143)
Employed	14.5 (9)	11.8 (6)	16.4 (11)	14.4 (26)
Unemployed	3.2 (2)	7.8 (4)	7.5 (5)	6.1 (11)

^a^Age presented as mean (s.d.). ^b^Sex assigned at birth. ^c^Includes multiple ethnicities or other (including Latino or Latin-American (*n* = 5), Indian (*n* = 2), Pakistani (*n* = 1), Chinese (*n* = 1), Turkish (*n* = 1), Albanian (*n* = 1) or not specified (*n* = 14)). GCSE, General Certificate of Secondary Education; NVQ, National Vocational Qualification

Source data

Table 2 reports the psychiatric history of mental health diagnosis within each group. The self-harm enaction group overall reported more history of a mental health diagnosis than the self-harm ideation group, with 64.2% reporting at least one mental health diagnosis compared to 34.3% in the self-harm ideation group. The enaction group was also more likely to report multiple diagnoses (40.3%) than the ideation group (25.5%). In both groups, depression and anxiety were the most commonly reported mental health problem.

**Table 2:** Rates of mental health diagnoses reported within the three self-harm groups (*n* = 180)

	Total sample (*n* = 180), *N* (%)	Control (*n* = 62), *N* (%)	Self-harm ideation (*n* = 51), *N* (%)	Self-harm enaction (*n* = 67), *N* (%)
Depression	51 (28.5)	0	14 (27.5)	37 (56.1)
ADHD	2 (1.1)	0	0	2 (3)
Problems with irritability or anger	2 (1.1)	0	1 (2)	1 (1.5)
Manic depression, mania or bipolar disorder	5 (2.8)	1 (1.6)	2 (3.9)	2 (3)
Anxiety disorders (including panic attacks)	43 (23.9)	0	14 (27.5)	29 (43.3)
Problems with alcohol or drugs	2 (1.1)	0	0	2 (3)
Any other emotional problems diagnosed	8 (4.7)	0	1 (2)	7 (11.9)
No. of diagnoses reported				
1	22 (12.2)	1 (1.6)	5 (9.8)	16 (23.9)
2	33 (18.3)	0	12 (23.5)	21 (31.3)
3	4 (2.2)	0	1 (2)	3 (4.5)
4	2 (1.1)	0	0	2 (3)
5	1 (0.6)	0	0	1 (1.5)

ADHD, attention deficit hyperactivity disorder (autism was not assessed).

Source data

Self-harm history for the self-harm enaction group is reported in Table 3. Self-harm was either non-suicidal (that is, non-suicidal self-harm) or suicidal (that is, a suicide attempt), as per the National Institute for Health and Care Excellence (NICE) definition of self-harm, which includes an episode regardless of the intent of the action. Most participants had a history of non-suicidal self-harm (92.25%), and nearly half (47.76%) had a history of both. Age of onset for both self-harm with and without suicidal intent was typically between the ages of 11 and 16 years. Most participants had carried out between two and ten acts of self-harm in the last year (61.29%), and one suicide attempt within the last year (28.6%). For non-suicidal self-harm, the method most often reported was cutting self (82.23%) and for a suicide attempt, overdose was most commonly reported (57.14%).

**Table 3:** History of self-harm behaviors (with and without suicidal intent) within the self-harm enaction group (*n* = 67)

	Self-harm (non-suicidal) (*n* = 62), *N* (%)	Suicide attempt (*n* = 35), *N* (%)
Age of onset, years		
<11	3 (4.90)	0
11–16	50 (81.90)	20 (57.20)
17–24	8 (13.10)	15 (42.80)
No. of episodes, lifetime		
1	1 (1.61)	21 (60.00)
2–10	16 (25.80)	14 (40.00)
11–50	18 (29.03)	0
51–100	21 (33.87)	0
101+	18 (29.03)	0
Too many to count	6 (9.68)	0
No. of episodes, last year		
1	9 (9.68)	10 (28.60)
2–10	38 (61.29)	2 (5.71)
11–50	5 (7.69)	0
51–100	3 (4.84)	0
101+	2 (3.22)	0
Too many to count	5 (7.69)	0
Primary method		
Cutting self	51 (82.23)	
Overdose		20 (57.14)
Hanging/strangulation		5 (14.29)
Multiple methods	40 (64.51)	2 (5.71)
Other	11 (17.77)	10 (28.57)

Note: *n* = 1 not sure age of onset; *n* = 32 history of both non-suicidal self-harm and suicide attempt. Age of onset has been collated into approximate education age ranges in the United Kingdom; primary school <11 years, secondary 11–16 years, and 17–24 years.

Source data

### Baseline analysis

Potential baseline differences in tonic SCL between the self-harm groups (control, self-harm ideation and self-harm enaction) were tested using a one-way analysis of variance (ANOVA). The results found no differences between the groups at baseline (*F*(2) = 1.704, *P* = 0.185, eta-squared (*η*^2^) = 0.019). Additionally, there were no significant differences between the groups on average SCR amplitude at baseline (*F*(2) = 0.60, *P* = 0.4349, *η*^2^ = 0.012). There were no differences by sex for baseline SCL (*F*(1) = 0.085, *P* = 0.771, *η*^2^ = 0.0005) or NS-SCRs (*F*(1) = 1.147, *P* = 0.286, *η*^2^ = 0.007). Correlation analysis found that baseline SCL and NS-SCR amplitudes are moderately positively correlated (*r* = 0.551, *P* < 0.001).

### Tone habituation analysis

Several participants (*n* = 10) did not produce an SCR to any of the tones. Previous studies have suggested that some people produce no or low SCRs, although rates of exclusion on this basis are not often reported^30^. These ten participants were excluded from the tone habituation analysis (*n* = 3 (4.8%) control group, *n* = 2 (4%) self-harm ideation group, *n* = 5 (7.5%) self-harm enaction group), and the final group numbers were *n* = 59 controls, *n* = 48 self-harm ideation and *n* = 62 self-harm enaction.

**Fig. 2:**
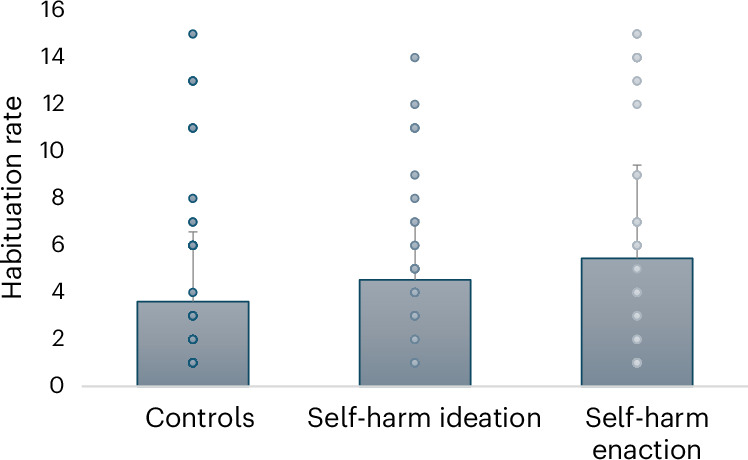
Poisson log-linear GLM testing differences in habituation rate to auditory tones (1–15) between the self-harm groups (*n* = 169). Note that the habituation rate considers the number of tones eliciting an SCR after threeconsecutive non-responses. χ^2^(2) = 30.41, P < 0.001, adjusting for age and sex. Controls: mean (s.d.) = 3.61 (2.97), n = 59; self-harm ideation: mean (s.d.) = 4.54(2.55), n = 48; self-harm enaction: mean (s.d.) = 5.46 (3.97), n = 62. Source data

Results from a Poisson log-linear generalized linear model (GLM) indicated that there were significant self-harm group differences in tone habituation rate (*χ*^2^(2) = 30.41, *P* < 0.001) when controlling for age and sex. Figure 2 displays these differences, showing that the self-harm enaction group reported the highest habituation rate (that is, number of SCRs before habituating to the tones), followed by the self-harm ideation group, then the control group. When inspecting the group differences more closely, it is evident that the self-harm enaction group had a higher habituation rate than the control group (odds ratio (OR) = 1.617; 95% confidence interval (CI) = 1.3608–1.923, *P* < 0.001) and than the self-harm ideation group (OR = 1.293; 95% CI = 1.090–1.533, *P* = 0.003).

### Psychosocial stress task analysis

Due to equipment problems during the Maastricht acute stress test (MAST), during which some participants’ EDA measures did not save during the stress task, five people were excluded (*n* = 1 (1.6%) in the control group, *n* = 0 (0%) in the self-harm ideation group and *n* = 4 (5.7%) in the self-harm enaction group). For this analysis, the final groups numbered *n* = 61 controls, *n* = 52 self-harm ideation and *n* = 66 self-harm enaction.

**Fig. 3:**
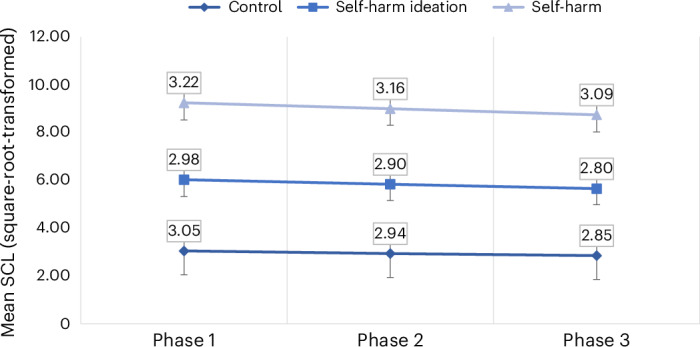
Differences in mean tonic SCL (square-root-transformed) between the self-harm groups during the phases of the psychosocial stress task (*n* = 177). Score range: minimum = 1.079, maximum = 5.159. Data labels report meanvalues. Error bars represent s.d. Controls: phase 1, n = 61, s.d. = 0.77; phase 2,n = 61, s.d. = 0.81; phase 3, n = 61, s.d. = 0.83. Self-harm ideation: phase 1, n = 51,s.d. = 0.70; phase 2, n = 51, s.d. = 0.68; phase 3, n = 51, s.d. = 0.67. Self-harmenaction: phase 1, n = 65, s.d. = 0.72; phase 2, n = 65, s.d. = 0.69; phase 3, n = 63,s.d. = 0.72. Source data

We ran two GLMs exploring the main effects for self-harm group, phase, sex and age, and the interaction of self-harm group and phase (Table 4 and Supplementary Table 3). Model 1 shows that there are significant group differences in SCL, with the self-harm enaction group exhibiting higher SCL than the control group (OR = 1.219 (1.055–1.410), *P* = 0.007) and self-harm ideation group (OR = 1.319 (1.135–1.534), *P* < 0.001). Figure 3 displays the mean SCL over each phase, showing that the self-harm enaction group displays consistently higher values than the other groups. The main model effect of phase was not significant (Table 4); despite this, the parameter estimates suggest that the first phase of the MAST indicates an overall higher SCL than the final phase (OR = 1.180 (1.019–1.367), *P* = 0.03), suggesting that overall SCL did reduce during the stress task, although this was not a large effect (Fig. 3). An effect of age was found (OR = 0.925 (0.903–0.948), *P* < 0.001), suggesting that SCL decreased as age increased. There was also an effect of sex, as males (mean = 3.13) had a higher SCL than females (mean = 2.94; OR = 1.168 (1.031–1.323), *P* = 0.015).

**Table 4:** GLM overall model effects and group × phase interaction for mean tonic SCL (square-root transformed) during the psychosocial stress task (*n* = 178)

Variable	Model 1, main effects *χ*^2^ (*P*)^a^	Model 2, group × time *χ*^2^ (*P*)
Group^b^	14.256 (<0.001)	14.279 (<0.001)
Phase^c^	4.8790 (0.087)	4.905 (0.086)
Age	39.244 (<0.001)	39.277 (<0.001)
Sex^d^	5.939 (0.015)	5.935 (0.015)
Group × phase		0.178 (0.996)
Observations (*n*)	529	529
Goodness of fit		
AIC^e^	1,144.496	1,152.318
BIC^f^	1,178.664	1,203.570
Omnibus test (*χ*^2^)^g^	59.568 (<0.001)	59.746 (<0.001)

^a^Wald estimate chi-squared test. ^b^Control, self-harm ideation, self-harm enaction. ^c^Phases of the MAST. ^d^Sex assigned at birth (female, male). ^e^Akaike’s information criterion. ^f^Bayesian information criterion. ^g^Compares the fitted model against the intercept-only model. Two-sided *P* value.

Model 2 included an interaction term between group and phase, testing whether there were differences between the self-harm groups over the phases of the stress task. As shown in Table 4, the interaction was not significant, suggesting there were no differences over the phases of the psychosocial stress task based on group membership. Additionally, the goodness of fit indices indicated that model 1 was a better fit for the data, suggesting that the interaction did not improve the model.

Correlation analysis suggested that the average SCR amplitude generated during the tones task was moderately positively correlated with SCL data during the psychosocial stress task (*r* = 0.352, *P* < 0.001), suggesting they are different but related constructs.

It should be noted that due to high levels of non-responding to the emotional images (i.e., no SCRs generated within 1–4 secs of image presentation), we had concerns about the robustness of this data and it was decided not to report these findings in the main body of the Article but to include in the Supplementary Information (Supplementary Table 4).

## Discussion

The current study investigated the extent to which EDA, a physiological index of emotion processing, distinguishes between young people who have no history of self-harm and those with a history of self-harm thoughts and those who have engaged in self-harm. We hypothesized that dysregulation of EDA may act as a volitional factor in distinguishing between thoughts of self-harm and self-harm acts. We probed this hypothesis using several established EDA measurements: (1) the SCR rate of habituation to auditory stimuli, (2) tonic SCL during a psychosocial stress task and (3) the average SCR amplitude in response to positive and negative images. The final task was not included in the main analysis because of high levels of non-response. Overall, the hypothesis was broadly supported, as in our sample of young people (16–25 years) those in the self-harm ideation group differed significantly from those in the self-harm enaction group in two of the primary EDA measurements. Specifically, we found that those in the enaction group took longer to habituate to the impersonal tones stimuli, and displayed a higher tonic SCL during the phases of the psychosocial stress task than those in the ideation group. No differences were found in the groups’ EDA responses to emotional images before and after the psychosocial stress task, although some potential issues were noted with this paradigm. Taken together, these findings suggest young people who self-harm may experience EDA hyperreactivity (that is, a heightened response to stimuli).

The finding that those in the self-harm enaction group exhibited a slower habituation rate (hyperreactivity) compared to the control and self-harm ideation groups is not consistent with some of the previous research investigating habituation of SCRs to auditory stimuli and suicide risk. This previous research suggests that those who have made a suicide attempt are more likely to demonstrate a faster habituation rate (hyporeactivity, or dampened response) compared to those who have not made a suicide attempt^31^, with further research investigating the operationalization of this marker in clinical settings to identify those at increased risk of suicide^32^. Several differences in our sample may explain some of these inconsistent findings. For example, we recruited a young sample (16–25 years) of participants who had engaged in or thought about self-harm, regardless of the intent of those actions, whereas most of the previous research on EDA habituation rate and suicide risk has been conducted with clinically depressed adults, who often had a history of a ‘violent’ suicide attempt^31^. Indeed, the majority of our sample had engaged in non-suicidal self-harm during the previous year, with only 12 making a suicide attempt. As self-harm serves a variety of functions, including regulation of emotions, and is not necessarily related to a desire to die^33^, the heterogeneity of motives may explain, in part, this inconsistency. As noted in the meta-analysis by Bellato and colleagues^28^, the type of self-harm appears to influence how the EDA dysregulation manifests. Specifically, studies of non-suicidal self-harm reported heightened EDA (that is, hyperreactivity) in young people who had self-harmed, whereas those that included suicidal ideation and behaviors found a lowered EDA response^28^. Although the studies in the meta-analysis included those using emotive or stressful stimuli, it may be that this distinction relating to suicidal or non-suicidal motives for self-harm also applies within the EDA habituation paradigm. This may reflect the underlying mechanisms that drive individuals to self-harm with and without suicidal attempt, in particular considering self-harm as a way to help to regulate a heightened autonomic response to various forms of stimuli. To our knowledge, our sample is younger than that which is typically investigated in EDA habituation studies, so, consistent with other physiological markers such as cortisol, where age effects are established, hyperreactivity may transition to hyporeactivity with age. Specifically, a meta-analysis found that the direction of the association between cortisol response and self-harm or suicide risk seems to reverse with age, with younger people eliciting higher cortisol levels and older individuals exhibiting lower cortisol in stress-reactivity paradigms^34^. This suggests that the physiological response to stress changes as those at risk of suicide age, possibly helping to explain the apparently contradictory findings in the EDA habituation literature, specifically that some studies have found that those at increased risk of suicide appear to elicit a dampened electrodermal response^31^, whereas the current study found an elevated EDA response (through slower habituation) in 16–25-year-olds to the auditory stimuli, suggesting a heightened physiological response.

Furthermore, the SCL data during the psychosocial stress task also showed that those in the self-harm enaction group exhibited a higher SCL during the task compared to both those in the control and self-harm ideation groups. This finding is consistent with some of the previous literature that found that young people who self-harm had higher physiological reactivity (skin conductance) during a distressing task^14^. However, other research found that low physiological arousal during stress in adolescents interacted with impulsivity to predict self-harm^23^, and during an emotional task, lower EDA was observed among participants with a history of self-harm compared to a depressed group^25^. These studies with inconsistent findings also utilized younger adolescent samples, and, rather than measure tonic SCL during the tasks, they appeared to measure non-specific SCRs. Accordingly, these different EDA measures may account for the inconsistencies. Additionally, we directly compared those who enacted self-harm with those who had thought about self-harm, and our findings suggest that young people who engage in self-harm have a different physiological response to stressful stimuli than those who think about self-harm, indicating that this may be a biomarker that could distinguish between these groups. It should be noted that the self-harm enaction group did have a more severe psychiatric history compared to the self-harm ideation group (that is, higher rates of depression and anxiety); although this is typical within self-harm research, it may be that some of the effects are related to diagnostic history. Placed within the context of the integrated motivational-volitional (IMV) model^11^, a predominant model of suicidality, heightened arousal of the electrodermal system seems to act as a volitional factor that may underpin the transition from thoughts to acts of self-harm.

No differences were found between the self-harm groups in SCRs to positive and negative images, although responding to the images was inconsistent and may reflect the issues around the robustness of this paradigm or with fatigue. The images may not have been sufficiently emotive to generate an SCR, particularly after the stress task when they were exposed to the same images. We suggest that future research may benefit from further exploration of emotive stimuli paradigms with young people who self-harm.

### Strengths and limitations

The current study has several strengths. First, it tested different paradigms in the investigation of electrodermal activity as a physiological proxy for emotion regulation in young people who self-harm, that is, the body’s natural physiological habituation to auditory stimuli and electrodermal activity during a psychosocial stress task. Second, the consideration of study design is important, as in previous research there have been inconsistent findings; importantly this may be related to age and sex differences, which our analysis has accounted for. Third, the study of physiological markers for self-harm is an under-researched area, and this study acts to remedy the relative dearth of literature on this topic with young people who have engaged in self-harm compared to those who report self-harm ideation only. The distinction between thought and enaction is important, as it is essential to determine which factors are implicated in the transition of thoughts of self-harm to enaction of self-harm, thus better informing the identification of and intervention with those at risk. To this end, this study suggests that the dysregulation of the electrodermal system is one such potential biomarker.

It should also be noted that the current study had several limitations. First, as a result of COVID-19, recruitment targets were not met, and there were challenges around recruiting equal numbers of males and females, especially to the self-harm enaction group. Additionally, we did not collect data on participants’ culture or geographic background, which may impact generalizability. Second, there is a lack of consensus in how to approach the measurement of EDA and the transformation of these measurements for analysis, with suggestions that all techniques have their own limitations^19^. In the current study, the data were square-root-transformed as this method was more effective in reducing the skew and kurtosis in the data, but some other studies have used logarithmic transformation^35^. Additionally, similar to other research, we found SCRs did not always occur linearly in response to each stimulus presentation, which may result in some selection bias^36^. Furthermore, many previous studies do not report exclusions due to non-responses to the stimuli^30^, and therefore we were unable to establish the extent to which our exclusion rates were comparable. The field should agree a consensus on how best to measure, transform and report EDA data. It should also be noted that the self-harm enaction group had a more severe psychiatric history than the self-harm ideation group; although this is typical within such studies, it may be that some of the effects are related to diagnostic history. Finally, we did not measure any other physiological indicators of the stress response (that is, heart rate or cortisol). However, as evidence suggests that stress induction may influence areas of the autonomic nervous system differently in those who self-harm^37^, future research should integrate these, alongside EDA, to determine the nature of these relationships. Despite these limitations, however, the current study adds considerably to the literature exploring dysregulation in physiological responses for those at risk of suicide.

### Clinical implications

The hyperreactivity evident in young people who self-harm may represent a dysregulated response to stressful and auditory stimuli, and as such may represent an underlying function of enacting self-harm, as the young person aims to regulate their stress response. A greater understanding of physiological mechanisms such as this could aid in the clinical interpretation of how and why self-harm manifests in some young people but not in others, and help inform how best this can be managed. This elevated response could also be used to potentially identify who may be at increased risk for self-harm, and to aid in the provision of early intervention to reduce self-harm risk. Although a potentially useful biomarker, it may be difficult to implement EDA measurements within clinical settings, so more consideration is required in terms of the implementation of such biomarkers clinically.

As EDA is proposed to be a physiological proxy of emotional arousal, its dysregulation reflects a heightened or dampened sympathetic activation, highlighting that how an individual copes with dysregulated arousal may be important for emotion regulation^35^. Evidence suggests that adolescents and adults who report poor emotion regulation also report higher suicidal ideation and suicide attempts^38^. However, emotion self-regulation strategies have been successful in modulating EDA, and this voluntary regulation of EDA is linked to cognitive reappraisal and the activation of prefrontal brain regions^39^. Cognitive reappraisal is a strategy that involves changing one’s interpretation of a situation to modulate its emotional impact, and the use of emotion-regulation strategies is associated with reduced autonomic arousal levels and EDA activity^22^. For example, while watching emotional film clips, participants’ SCL responses were found to be influenced by emotion-suppression strategies^40^, and those exhibiting high levels of anxiety around maths used cognitive reappraisal techniques to reduce the negative association between higher physiological arousal and poorer accuracy in maths^36^. Therefore, emotion-regulation strategies may be important in regulating EDA and potentially for self-harm management.

Another potential application of the findings is to utilize an individual’s EDA as a means of recognizing how the body responds to emotional arousal and then employ techniques to adapt this response. For example, biofeedback may be useful in this regard. Biofeedback is a non-invasive bio-behavioral approach where an individual trains to achieve volitional control over an autonomous bodily process; with evidence that biofeedback interventions (primarily using electroencephalographic neurofeedback) can reduce symptoms in clinical populations^41^. Other studies have used EDA biofeedback, by using biofeedback to successfully attempt to manipulate the sympathetic activation of the skin that is associated with emotional arousal^42^. Indeed, EDA biofeedback training has been used as a treatment for managing eating disorders^43^, anxiety/stress^44^ and depersonalization disorder^45^. This strategy may be adapted for self-harm, for example, as an additional non-invasive treatment for young people who self-harm to recognize, monitor and control their dysregulated physiological arousal.

## Conclusions

There is a need to better understand the factors associated with self-harm in young people, with greater attention on the physiological biomarkers of self-harm risk long overdue. The current study suggests that young people who have a history of self-harm exhibit a heightened electrodermal response to both stressful and non-stressful stimuli compared to those who have no history of self-harm and those who have only thought about self-harm. Therefore, this finding advances our understanding of a key physiological mechanism, or biomarker, potentially underpinning the transition from thoughts to acts of self-harm. Clinically, this could help us to better identify young people who are at higher risk of self-harm, and to inform the development of future basic science and treatment studies.

## Methods

### Participants and recruitment

This study adopted a quasi-experimental approach, as groups were predefined and not randomized^46^. Ethical approval was granted from the University of Glasgow’s College of Medical, Veterinary & Life Sciences (MVLS) ethics board (200180180). The study was not preregistered, but an analysis plan was submitted with the grant application.

Three groups of participants were recruited: a control group with no self-harm history, a self-harm ideation group with a history of thoughts, but no enactment of self-harm ever, and a self-harm enaction group who had harmed themselves within the past 12 months. A flow chart outlining those potential participants who were excluded during screening and recruitment is displayed in Fig. 1. After exclusions due to non-response (*n* = 3), equipment problems (*n* = 3) or self-harm history (*n* = 1; seven excluded in total, *n* = 2 control, *n* = 2 self-harm ideation, *n* = 3 self-harm enaction), the final group numbers were: *n* = 62 control, *n* = 51 self-harm ideation and *n* = 67 self-harm enaction groups.

A full breakdown of the demographic characteristics of each group is outlined in Table 1. Sex was self-reported as ‘sex assigned at birth’, due in part to the biological nature of the study. Ethnicity was self-reported. It should be noted that recruitment was adversely affected by COVID-19, as it was halted as a result of the lockdown periods, and it was slower than we anticipated when the COVID-19 restrictions were eased and then lifted. Participant recruitment was delayed because of COVID-19, with recruitment beginning following the final COVID-19 lockdown in July 2021. Some participants (*n* = 19) were recruited when wearing face coverings in public places (including classrooms) was a legal requirement in Scotland (this restriction was lifted on 18 April 2022). Throughout recruitment, participants were informed that masks were optional, but it was not recorded whether they wore a face covering or not.

Participants were recruited using a range of approaches, including via online advertisements (for example, social media), advertisements placed in the community (for example, local colleges, psychology participant pool) and by contacting relevant organizations to share the study details (for example, Penumbra self-harm network, Bipolar Scotland, MQ Mental Health). Those who expressed an interest in the study completed a short online screening tool assessing their eligibility, which included questions about their self-harm history and health conditions that may make them ineligible for the cold pressor test and the physiological measures (for example, heart conditions, diabetes, epilepsy, Reynaud’s syndrome), and provided their contact details and a suitable contact time. Researchers then contacted potentially eligible participants by telephone to complete a brief screening, where their current self-harm and mental health history was assessed. Self-harm ideation was assessed with the question ‘Have you ever had *thoughts* of purposely hurting yourself whether or not you wanted to die?’ and self-harm enaction was assessed with ‘Have you ever hurt yourself purposely whether or not you wanted to die?’. Both were followed with ‘If so, when was the last time?’. Participants were eligible to take part if they had no history of self-harm ideation or enaction or any mental health diagnosis (controls), if they had self-harm ideation in the past 12 months but had never enacted (self-harm ideation group) and if they had enacted self-harm in the past 12 months (self-harm enaction). When a participant met the eligibility criteria, an appointment was made for the participant to visit the Mindstep Health Lab at the University of Glasgow. Those who took part received £30 as compensation for their time. Anyone ineligible to take part (through the online or phone screening) was thanked for their time and sent a support sheet listing the contact details of relevant organizations. Those who expressed recent self-harm ideation or behaviors were asked about their current desire to live, and a risk assessment and safety plan was conducted if they were deemed at risk.

### Procedure

During the lab appointment (Supplementary Fig. 1 provides the study flow chart), participants were asked to read an information sheet and then signed a consent form to agree to take part in the study. They were told they could stop participation at any time without giving a reason. The experimental procedure included several phases: (1) a familiarization and baseline measurement phase, (2) a tonal habituation task phase, (3) an emotional images task phase and (4) a psychosocial stress task phase. Finally, participants were interviewed to assess their history of mental health disorders and self-harm (including with and without suicidal intent).

### Electrodermal activity recording

Participants were familiarized with the EDA recording equipment. Two surface Ag/AgCl disposable electrodes were attached to the participants’ non-dominant hand (distal phalanges of the first and second fingers) to measure EDA throughout the experiment. The units used for measuring EDA electrical conductance are microsiemens (μS), with typical skin conductance levels in the range of 2–20 μS (ref. ^19^). For data acquisition, a BIOPAC MP160 module with an EDA100C-MRI Smart Amplifier was linked to a laptop using AcqKnowledge (version 5.0.5) software to process the EDA signal (Biopac Systems). The sampling rate was 25 Hz and the gain 2 μS V^−1^, the low-pass filter was set at 1 Hz and the high-pass filter at 0.05 Hz.

### Baseline measurements

After familiarization with the EDA equipment, baseline recordings (3 min) of average SCL and the average amplitude of the NS-SCR were taken while participants viewed a neutral image (a black X on a white background). During this time, participants were instructed to rest their hand with the EDA electrodes in a supine position on the armrest and to move it as little as possible.

### Tonal habituation task

This task uses impersonal tones to elicit the participants’ natural SCRs to auditory stimuli. Based on a similar procedure to that outlined in ref. ^29^, a series of 15 moderately loud sinus tones (80 dB, 1 kHz, 1-s duration) at varying interstimulus intervals (15, 20 and 25 s) were administered to the participants via headphones in a sequence that appeared to be random to the participant. An SCR to a tone must occur within 1–4 s after tone onset and have a minimum amplitude of 0.05 μS. Consistent with previous research^15,29^, the habituation rate was the number of the stimulus (that is, 1–15 tones) that produced the last SCR amplitude, and where no other SCR had been detected over three subsequent stimuli. Using AcqKnowledge (version 5.0.5) software, SCRs were identified and exported to Excel, and habituation rates were calculated (range 1–15).

### Psychosocial stress task

The MAST^47^ was used to stimulate physiological stress responses. It includes five socially evaluated cold pressor trials where participants immerse their dominant hand in an ice-cold water bath for varying durations (60–90 s) over a 10-min time span. Between trials, participants are instructed to perform mental arithmetic as quickly and as accurately as possible, and receive negative feedback on their performance when mistakes are made. To heighten the social evaluation component, participants are falsely informed that they are being videotaped throughout for facial expression analyses. Throughout the stress task, consistent with previous research, SCLs are measured^14,27^, and data from three time points are extracted to determine differences in the pattern of electrodermal responding. Specifically, average SCLs are measured at three time periods (epochs lasting 90 s) at the start, middle and end of the task to establish how SCL changes over the course of the stress task for each group. The data were exported and epochs derived using PhysioData Toolbox (version 0.6.3)^48^ analyzers, which allow for the SCL signal to be low-pass-filtered (with shock removal) and smoothed by resampling the filtered signal with a 20-Hz time vector. The typical range for tonic SCL is between 2 μS and 20 μS (ref. ^35^). To be conservative we excluded individuals with an average SCL below 1 μS, with one participant excluded based on this criterion.

### Emotional images

Participants were exposed to a series of 21 images selected from the International Affective Picture System (IAPS)^49^, before and after the psychosocial stress task (MAST). The images are designed to create an emotional response, and images from each of the valence (positive, neutral, negative) × arousal (high, low) categories were chosen. Images were presented in a randomized order to each participant at each time point. Like the tones task, any SCR that occurred within 1–4 s of image presentation was recorded as an EDA response to the image. An average SCR amplitude was calculated for positive, negative and neutral images at both time points, to assess whether the stress task had an impact upon responses to the images.

### Diagnostic and self-harm history interview

Psychiatric history was assessed with a question directly asking whether they had ever received a mental health diagnosis. If yes, participants were given options for their diagnosis and could select as many as they felt applied: depression, ADHD, problems with irritability or anger, manic depression, mania or bipolar disorder, anxiety disorders (including panic attacks), problems with alcohol or drugs and any other emotional problems.

Self-harm history was assessed using the Self-Injurious Thoughts and Behaviours Interview (SITBI)^50^, a structured interview used to assess the presence, frequency and characteristics of self-injurious thoughts and behaviors, including suicidal ideation, suicidal attempts and non-suicidal self-injury. The SITBI is a widely used measure demonstrating good interrater reliability, test–retest reliability and concurrent validity with young people^50^.

### Statistical analysis

All analysis was conducted using SPSS version 29^51^. No missing data were imputed. De-identified data and code for the current analysis has been made available on the Open Science Framework (https://osf.io/g8ejf/). The literature suggests that SCL tonic data should be transformed to reduce skew and kurtosis in the data and adjust for individual differences^19^. A distribution is approximating normality if skewness or kurtosis (excess) values are between −1 and +1 (ref. ^52^). The literature recommends that logarithmic or square-root transformations are suitable to be applied to SCL data^35^. Although the data did not indicate extreme skew, some kurtosis was evident (Supplementary Table 1), and it was decided to transform the data as per the recommendations in the literature. Upon performing both logarithmic and square-root transformations, we found that the skew and kurtosis values better approximate normality with the square-root transformation (Supplementary Table 1). Therefore, SCL data were square-root-transformed, which had the additional benefit of removing some of the individual differences in SCL within the data, and guidance suggests that further adjustment for individual differences can be problematic and may not be necessary^19^. A similar procedure was applied to the SCR data in the emotional images task. Furthermore, a sensitivity analysis suggested that the results were similar, regardless of the transformation adopted (Supplementary Table 2).

### Baseline measurement

We tested for initial baseline differences within the SCL tonic and NS-SCRs (that is, SCRs generated in the absence of stimuli) amplitude data to identify whether there were any individual level differences between the groups before any stimuli presentation, using one-way ANOVA; where applicable, Bonferroni post hoc corrections would also be applied. Sex (assigned at birth; male, female) differences in baseline SCL and NS-SCR amplitudes were also investigated using one-way ANOVA. Furthermore, we conducted a correlational analysis to establish whether baseline SCL and NS-SCR amplitude data were significantly associated and in which direction.

### Auditory tones task

The outcome measure for the tones task was the habituation rate (range 1–15) for each participant, that is, the number of tones eliciting an SCR within 1–4 s, before three consecutive non-responses. GLM was used to test for differences between the self-harm groups in the tones task habituation rate, and analysis was adjusted for sex and age. GLM is an umbrella term that encompasses several models, expanding the general linear model so that the dependent variable is linearly related to the factors and covariates via a specified link function. This allows for the outcome variable (*Y*) to have an error distribution other than a normal distribution; it extends linear models to allow count variables, and it is useful when the data are clustered, for example, repeated observations of the same participants^53^. The output for models includes goodness-of-fit statistics (AIC and BIC), model effects (chi-squared) and parameter estimates, such as ORs. For the tone habituation task analysis, a Poisson log-linear model was selected, where the Poisson distribution is the number of occurrences of an event of interest, and the log link function transforms the count variable for analysis.

### Psychosocial stress task

To test for self-harm group differences during the MAST, filtered SCL tonic data from three 90-s phases from the start, middle and end of the task were extracted using PhysioData Toolbox. The overall mean SCL during these phases of the MAST was calculated, and the self-harm groups were compared using multilevel linear GLM with the identity link function (as data have already been transformed). Within the models we included the covariates of age, sex and phase (three levels) of the task. Interaction for the self-harm groups with phase of the task was added to the GLM in a further model. The overall model effects for the variables and interactions are reported for each model in Table 4, and the ORs for each of the categorical variables in relation to tonic SCL are reported in Supplementary Table 3. Finally, we conducted a correlation analysis to establish the association between the SCR amplitudes generated during the tones task and the SCL during the phases of the stress task.

### Emotional images task

An image must elicit an SCR within 1–4 s of image presentation, and the mean SCR amplitude in response to both positive (*n* = 7) and negative (*n* = 7) images was calculated. As with previous measurements, SCR amplitudes were square-root-transformed. GLM was used to test for differences between the self-harm groups in the mean SCR amplitude in response to negative and positive images, and this included a time variable for before and after the psychosocial stress task. A linear model was selected, because the SCR amplitude is a continuous variable. All analyses were adjusted for sex and age. However, there were high levels of non-responding, that is, participants not generating any SCRs, or only one or two SCRs, to the negative or positive images, and as we use an average SCR as the outcome, we were concerned about the robustness of these data. Specifically, post the stress task, *n* = 67 (37%) participants generated fewer than two SCRs to the 14 positive and negative images (a 14.2% response rate). Often, responses were not generated to either negative or positive images, so calculating an average score was compromised. It may be that the images were not sufficiently emotive, particularly given the repetition after the stress task. Consequently, we decided not to report these findings in the main body of the Article but to include these analyses in the Supplementary Information (Supplementary Table 4). For the analysis, in the interests of completeness, we included all participants who elicited at least one SCR to a positive or negative image (*n* = 131), and, on performing a sensitivity analysis, excluding those who did not respond to more than one image (*n* = 110), we found the results were the same.

### Power calculation

Using a power calculation, based on previous research^14^, with an effect size of Cohen’s *d* = 0.47, with 0.80 power, alpha set at 0.05 and assuming a within participants correlation of 0.50, it was calculated that we would need 72 participants per group to identify an interaction between group and time. As noted, this was not achieved for each group, which may suggest that the interaction analysis may be underpowered. However, to identify main effects, a sample size of 52 participants was required, so there was likely sufficient power to identify main effects.

### Reporting Summary

Further information on research design is available in the Nature Portfolio Reporting Summary linked to this article.

## Supplementary information

Supplementary Information Supplementary Fig. 1, Tables 1–4 and findings for the emotional images task.

Reporting Summary

## Source data

Source Data Fig. 2 Source data in Excel format.

Source Data Fig. 3 Source data in Excel format.

Source Data Table 1 Source data in Excel format.

Source Data Table 2 Source data in Excel format.

Source Data Table 3 Source data in Excel format.

## Data Availability

De-identified data for this paper have been made available on the Open Science Framework (https://osf.io/g8ejf/).

## Code availability

No custom code was developed for this paper.
