# Supplementary material for: Emotion processing and electrodermal activity in young people who self-harm
Source: Nat Ment Health. 2025 Nov 5;3(11):1374–83. doi: 10.1038/s44220-025-00520-5 (PMC12589109; doi:10.1038/s44220-025-00520-5)
Supplement: Supplementary file 1 — Supplementary Fig. 1, Tables 1–4 and findings for the emotional images task. [file 44220_2025_520_MOESM1_ESM.pdf]

nature mental health

Supplementary information https://doi.org/10.1038/s44220-025-00520-5

Emotion processing and electrodermal activity in young people who self-harm

In the format provided by the authors and unedited

Supplementary materials

Figure S1: Flowchart outlining the study design and procedure

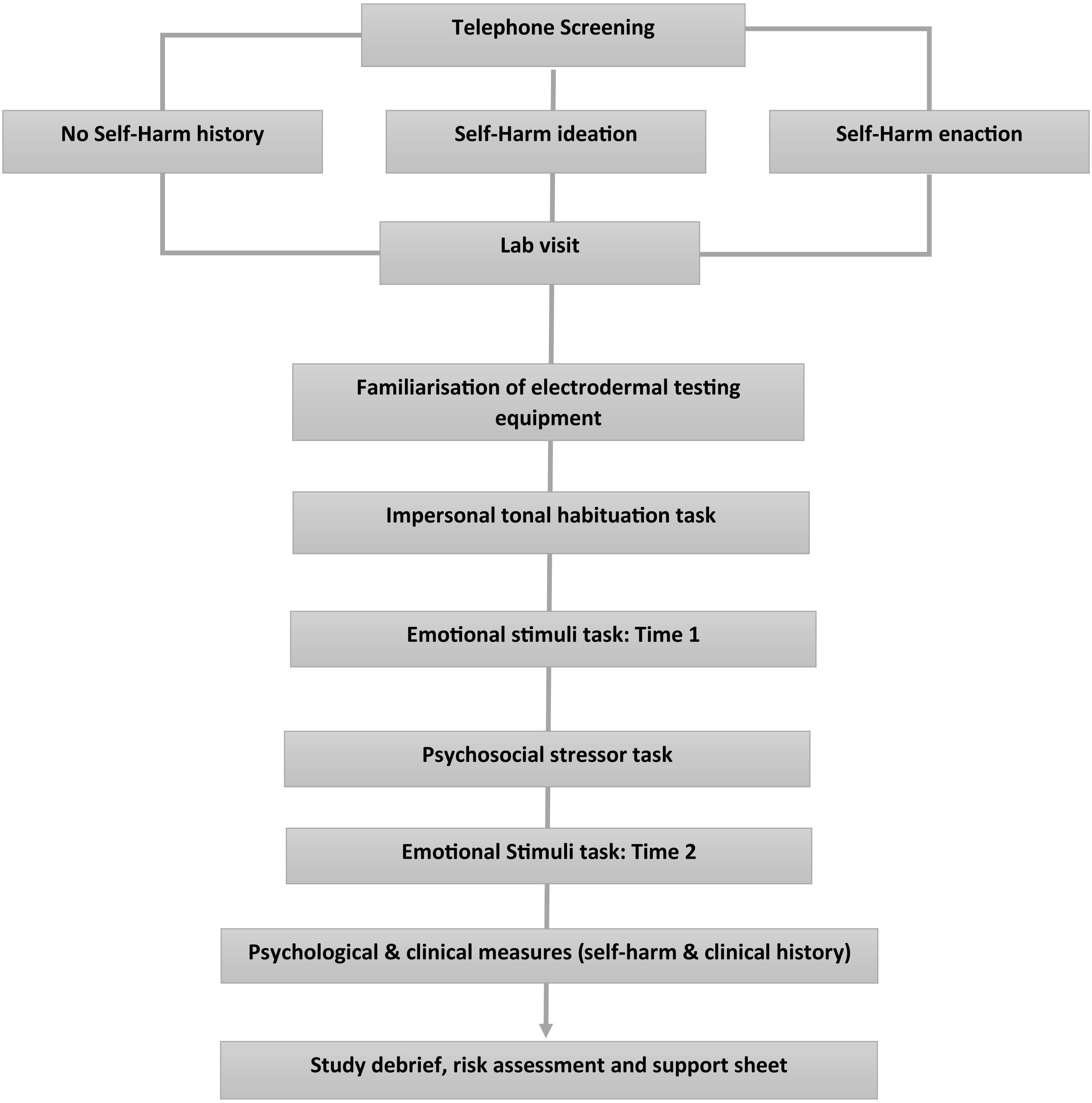

Table S1. Descriptive statistics for the SCL tonic data during the 3 phases of the MAST stress task, showing the skew and kurtosis before and after transformation

	N	Minimum	Maximum	Mean	Std. Deviation	Variance	Skewness	Std. Error	Kurtosis	Std. Error
**MAST_T1 _mean[1]**	179	1.16327	25.96590	10.11896	4.54603935	20.666	0.741	0.182	1.185	0.361
**MAST_T2_mean[1]**	179	1.16516	26.16502	9.615483	4.48901145	20.151	0.811	0.182	1.335	0.361
**MAST_T3_mean[1]**	176	1.13738	26.61782	9.119785	4.47875893	20.059	0.905	0.183	1.760	0.364
**MAST_T1_mean_sqrt[2]**	179	1.079	5.096	3.09616	0.731930	0.536	-0.119	0.182	0.488	0.361
**MAST_T2_mean_sqrt[2]**	179	1.079	5.115	3.01251	0.737100	0.543	-0.054	0.182	0.384	0.361
**MAST_T3_mean_sqrt[2]**	176	1.066	5.159	2.92512	0.752785	0.567	-0.015	0.183	0.360	0.364
**MAST_T1_mean_log[3]**	179	0.07	1.41	0.9540	0.23020	0.053	-1.165	0.182	2.404	0.361
**MAST_T2_mean_log[3]**	179	0.07	1.42	0.9286	0.23598	0.056	-1.018	0.182	1.623	0.361
**MAST_T3_mean_log[3]**	176	0.06	1.43	0.8999	0.24829	0.062	-0.954	0.183	1.186	0.364

[1] Before data SCL data is transformed, [2] SCL data after square root transformation, [3] SCL data logarithmic transformation

Table S2: Generalized Linear Models (GLM) with SCR data during stress task log transformed

**Variable**	**Model main effects (χ^2), p**
Group[1]	15.005, <0.001
Phase[2]	4.881
Age	30.734, <0.001
Sex[3]	6.629, 0.014

Wald estimate Chi-square, [1] Control, Self-harm ideation, Self-harm enaction, [2] Phases of the MAST, [3] Sex assigned at birth (female, male)

Table S3: Generalized Linear Models (GLM): parameter estimates for each variable in relation to the mean tonic SCL (square root transformed) during the psychosocial stress task (n=178)

Variable	Group	**Model 1: main effects, OR [95%CI], p**	**Model 2: group x time OR [95%CI], p**
Group[a]	Control	0.802 [0.709-0.948], 0.002	0.795 [0.620-1.020], 0.043
	SH Ideation	0.758 [0.652-0.881], <0.001	0.738 [0.569-0.958, 0.011
Sex[b]	Female	0.856 [0.756-0.970], 0.003	0.856 [0.756-0.958], 0.003
Age		0.925 [0.903-0.948], <0.001	0.925 [0.903-0.948], <0.001
Phase[c]	Phase 1	1.180 [1.019-1.367], 0.028	1.135 [0.890-1.449], 0.329
	Phase 2	1.086 [0.937-1.257], 0.269	1.069 [0.838-1.363], 0.614
Group x Phase[d]	Control x Phase 1		1.073 [0.903-1.521], 0.680
	Control x Phase 2		1.022 [0.721-1.448], 0.891
	SH Ideation x Phase 1		1.050 [0.729-1.514], 0.744
	SH Ideation x Phase 2		1.029 [0.714-1.483], 0.831

Reference Group: [a] SH enaction, [b] Male, [c] Phase 3, [d] SH enaction x Phase 3. Controls: Phase 1 n=61 SD=0.77, Phase 2 n=61, SD=0.81, Phase 3 n= 61, SD=0.83; SH Ideation: Phase 1 n=51, SD=0.70, Phase 2 n=51, SD=0.68, Phase 3 n=51, SD=0.67; SH Enaction: Phase 1 n=65, SD=0.72, Phase 2 n=65, SD=0.69, Phase 3 n=63, SD=0.72.

Emotional Images Results

Four GLMs were executed to test for self-harm group differences in the EDA response to both negative and positive images pre- and post- psychosocial stress task. Models included age and sex as covariates. The main effects and interaction between group X time was tested for separately, for both positive and negative images. Time 1 had more responses (n=146) than time 2 (n=131), but all responses were included in the multilevel model. The results of these models are reporting in Table S3.

Table S4: Generalized Linear Models (GLM): main effects and interactions for the average SCR's (square root transformed) elicted to positive and negative images before and after the psychosocial stress task (n=146)

**Variable**	**Negative images**		**Positive images**	
	**Model 1: main effects (χ^2)[a]**	**Model 2: group x time (χ^2)**	**Model 3: main effects (χ^2)[a]**	**Model 4: group x time (χ^2)**
Group[1]	0.928	0.959	0.935	0.855
Time [2]	32.344	32.253	10.060	9.359
Age	2.334	2.285	1.524	1.527
Sex[3]	2.079	2.032	1.282	1.301
Group*time		0.372		1.362
Observations (n)	277	277	277	277
**Goodness of fit**				
AIC[4]	225.197	228.825	191.321	193.961
BIC[5]	250.565	229.499	216.689	226.573
Omnibus Test (χ2)[6]	36.738	37.110	14.343	15.702

Notes: [a] Wald estimate Chi-square, [1] Control, Self-harm ideation, Self-harm enaction, [2] Pre- and post-stress task, [3] Sex assigned at birth (female, male), [4] Akaike's Information Criterion, [5] Bayesian Information Criterion, [6]Compares the fitted model against the intercept-only model.

Findings

Negative images

In model 1 there were no self-harm group differences found for the overall mean SCR responses to the negative images, as reported in Table S3. An effect of time was found, with a significant decrease in the size of the mean SCR amplitudes from time 1 (pre-stress task mean = 0.6907) to time 2 (post stress task mean = 0.4478; OR = 1.275 [1.172-1.386], p <0.001). No differences for age and sex were found. Model 2 found the interaction between group and time was not significant.

Positive images

As reported in Table S3, Model 3 found no self-harm group differences found for the overall mean SCR responses to the positive images. Additionally, there were no differences for sex and age found on mean SCR amplitudes. An effect of time was found, with a significant decrease in the size of the mean SCR amplitudes from time 1 (pre-stress task mean = 0.6287) to time 2 (post stress task mean = 0.5005; OR = 1.136 [1.050-1.230], p =0.002). Model 4 found the interaction between group and time was not significant.

A sensitivity analysis was conducted removing all participants who only responded to 1 image, as this may compromise the accuracy of the average measurement, it was found that results remained the same.
