# Supplementary material for: Emotion processing and electrodermal activity in young people who self-harm
Source: Nat Ment Health. 2025 Nov 5;3(11):1374–83. doi: 10.1038/s44220-025-00520-5 (PMC12589109; doi:10.1038/s44220-025-00520-5)
Supplement: Supplementary file 2 — Reporting Summary [file 44220_2025_520_MOESM2_ESM.pdf]

nature portfolio

Corresponding author(s): Dr Karen Wetherall

Last updated by author(s): Jul 1, 2025

Reporting Summary

Nature Portfolio wishes to improve the reproducibility of the work that we publish. This form provides structure for consistency and transparency in reporting. For further information on Nature Portfolio policies, see our Editorial Policies and the Editorial Policy Checklist.

Statistics

For all statistical analyses, confirm that the following items are present in the figure legend, table legend, main text, or Methods section.

n/a	Confirmed	
☐	☒	The exact sample size (*n*) for each experimental group/condition, given as a discrete number and unit of measurement
☐	☒	A statement on whether measurements were taken from distinct samples or whether the same sample was measured repeatedly
☐	☒	The statistical test(s) used AND whether they are one- or two-sided<br>*Only common tests should be described solely by name; describe more complex techniques in the Methods section.*
☐	☒	A description of all covariates tested
☐	☒	A description of any assumptions or corrections, such as tests of normality and adjustment for multiple comparisons
☐	☒	A full description of the statistical parameters including central tendency (e.g. means) or other basic estimates (e.g. regression coefficient) AND variation (e.g. standard deviation) or associated estimates of uncertainty (e.g. confidence intervals)
☐	☒	For null hypothesis testing, the test statistic (e.g. *F*, *t*, *r*) with confidence intervals, effect sizes, degrees of freedom and *P* value noted<br>*Give P values as exact values whenever suitable.*
☐	☒	For Bayesian analysis, information on the choice of priors and Markov chain Monte Carlo settings
☒	☐	For hierarchical and complex designs, identification of the appropriate level for tests and full reporting of outcomes
☐	☒	Estimates of effect sizes (e.g. Cohen's *d*, Pearson's *r*), indicating how they were calculated

Our web collection on statistics for biologists contains articles on many of the points above.

Software and code

Policy information about availability of computer code

Data collection	AcqKnowledge (version 5.0.5) software was used to to process the EDA signal (Biopac Systems Inc., Goleta, CA, USA). No other software or code was used to collect the data.
Data analysis	The skin conductance levels data were exported and epochs derived using PhysioData Toolbox (Version 0.6.3; Sjak-Shie, 2022). Analysis was conducted using SPSS version 29 (IBM Corp, 2023) and no original code was generated.

For manuscripts utilizing custom algorithms or software that are central to the research but not yet described in published literature, software must be made available to editors and reviewers. We strongly encourage code deposition in a community repository (e.g. GitHub). See the Nature Portfolio guidelines for submitting code & software for further information.

Data

Policy information about availability of data

All manuscripts must include a data availability statement. This statement should provide the following information, where applicable:

- Accession codes, unique identifiers, or web links for publicly available datasets
- A description of any restrictions on data availability
- For clinical datasets or third party data, please ensure that the statement adheres to our policy

De-identified data for the current analysis has been made publicly available on the Open Science Framework (https://osf.io/g8ejf/).

Research involving human participants, their data, or biological material

Policy information about studies with human participants or human data. See also policy information about sex, gender (identity/presentation), and sexual orientation and race, ethnicity and racism.

Reporting on sex and gender	Sex was self-report, Participnats were asked 'What was your sex assigned at birth?', due in part to the biological nature of the study it was decided to focus on biological sex rather than gender. Sex is reported in the main demographics table. Sex was added as a covariate in all analysis.
Reporting on race, ethnicity, or other socially relevant groupings	Ethnicity was self-reported, and assessed with this question: 'What is your ethnic group?'<br>Due to very small numbers of non-white or Asian participants, in the demographics table this was reduced to: Asian/British Asian, White and Other. Ethnicity was not included as a covariate.<br>Education and employment were also reported.
Population characteristics	See below. Continuous age was included as a covariate in all analysis and is reported in the main demographics table
Recruitment	Participants were recruited using a range of approaches, including via online advertisements (e.g., social media), advertisements placed in the community (e.g., local colleges, psychology participant pool) and by contacting relevant organisations to share the study details (e.g., Penumbra self-harm network, Bipolar Scotland, MQ Mental Health). Those who expressed an interest in the study completed a short online screening tool assessing their eligibility, which included questions about their self-harm history and health conditions that may make them ineligible for the cold pressor test and the physiological measures (e.g., heart conditions, diabetes, epilepsy, Reynaud's syndrome). Bias in recruitment may be from exposure to the desired population, as not everyone who has a history of self-harm will have been approached.
Ethics oversight	Ethical approval was granted from the University of Glasgow's College of Medical, Veterinary & Life Sciences (MVLS) ethics board (200180180).

Note that full information on the approval of the study protocol must also be provided in the manuscript.

Field-specific reporting

Please select the one below that is the best fit for your research. If you are not sure, read the appropriate sections before making your selection.

☐ Life sciences ☒ Behavioural & social sciences ☐ Ecological, evolutionary & environmental sciences

For a reference copy of the document with all sections, see nature.com/documents/nr-reporting-summary-flat.pdf

Behavioural & social sciences study design

All studies must disclose on these points even when the disclosure is negative.

Study description	Quasi-experimental case control study (quantative data)
Research sample	In total, n=180 18-25 year olds took part in the study, demographics included: mean age was 21 years, 60% female, 50% White, 56.7% degree educated. The sample was not representative of the broader population, as specific self-harm histories were recruited to test for differences in physiological responses.<br>Three groups of young people (18-25 years) were recruited: a control group with no self-harm (SH) history, a self-harm (SH) ideation group with a history of thoughts, but no enactment of SH ever, and a self-harm (SH) enaction group who had harmed themselves within the past 12 months. The final group numbers were: n=62 control, n=51 SH ideation, n=67 SH enaction groups.
Sampling strategy	Convienence sampling was used as we used adverts online and in the community. Using a power calculation, based upon previous research (Nock & Mendes, 2008), with an effect size of d = .47, with .80 power, alpha set at .05 and assuming a within participants correlation of .50, it was calculated that we would need 72 participants per group to identify an interaction between group and time. As noted, this was not achieved for each group, which may indicate that the interaction analysis may be underpowered. However, to identify main effects, a sample size of 52 participants was required, therefore, was likely sufficient power to identify main effects.
Data collection	Written informed consent was obtained from all participants prior to study commencement.<br>EDA recording equipment. Two surface Ag/AgCl disposable electrodes were attached to the participants' non-dominant hand (distal phalanges of the first and second fingers) to measure electrodermal activity (EDA) throughout the experiment. The units used for measuring EDA electrical conductance are microsiemens (µS), with typical skin conductance levels in the range of 2-20 µS (Braithwaite et al., 2015). For data acquisition, a BIOPAC MP160 module with an EDA100C-MRI Smart Amplifier was linked to a laptop using AcqKnowledge (version 5.0.5) software to process the EDA signal (Biopac Systems Inc., Goleta, CA, USA). The sampling rate was 25 Hz, gain 2 µS/V, low pass filter set at 1 Hz and a high pass filter set to 0.05 Hz.<br><br>Experimenter was not blind. They were in the room with the participant for the duration. Diagnosis and suicide history data was collected by the experimenter using pen and paper and this data was later processed.
Timing	Start: 13.12.2021<br>End: 31.08.23

Data exclusions	7 participants were excluded from the analysis. 3 were excluded as they did not generate an electrodermal response as suggested by the literature, 3 due to equipment problems and 1 became ineligible after their self-harm history was attained in interview. A flow diagram is included in the paper.
Non-participation	19 people did not attend their appointment. Reason for non-participation is not known.
Randomization	Participants were allocated to a study group based on their self-harm history.

Reporting for specific materials, systems and methods

We require information from authors about some types of materials, experimental systems and methods used in many studies. Here, indicate whether each material, system or method listed is relevant to your study. If you are not sure if a list item applies to your research, read the appropriate section before selecting a response.

Materials & experimental systems

n/a	Involved in the study
☒	☐ Antibodies
☒	☐ Eukaryotic cell lines
☒	☐ Palaeontology and archaeology
☒	☐ Animals and other organisms
☒	☐ Clinical data
☒	☐ Dual use research of concern
☒	☐ Plants

Methods

n/a	Involved in the study
☒	☐ ChIP-seq
☒	☐ Flow cytometry
☒	☐ MRI-based neuroimaging

Plants

Seed stocks	*Report on the source of all seed stocks or other plant material used. If applicable, state the seed stock centre and catalogue number. If plant specimens were collected from the field, describe the collection location, date and sampling procedures.*
Novel plant genotypes	*Describe the methods by which all novel plant genotypes were produced. This includes those generated by transgenic approaches, gene editing, chemical/radiation-based mutagenesis and hybridization. For transgenic lines, describe the transformation method, the number of independent lines analyzed and the generation upon which experiments were performed. For gene-edited lines, describe the editor used, the endogenous sequence targeted for editing, the targeting guide RNA sequence (if applicable) and how the editor was applied.*
Authentication	*Describe any authentication procedures for each seed stock used or novel genotype generated. Describe any experiments used to assess the effect of a mutation and, where applicable, how potential secondary effects (e.g. second site T-DNA insertions, mosiacism, off-target gene editing) were examined.*
